# Supplementary material for: Allele-Specific Gene Editing Rescues Pathology in a Human Model of Charcot-Marie-Tooth Disease Type 2E
Source: Front Cell Dev Biol. 2021 Aug 16;9:723023. doi: 10.3389/fcell.2021.723023 (PMC8415563; doi:10.3389/fcell.2021.723023)
Supplement: Supplementary file 2 [file Data_Sheet_2.docx]

**Supplementary Table 1: Oligonucleotide Sequences**

| **Name** | **Sequence (5’ → 3’)** |
| --- | --- |
| hNIL AAVS1 5’ Junction F | CCTGAGTCCGGACCACTTTG |
| hNIL AAVS1 5’ Junction R | AGAAGACTTCCTCTGCCCTC |
| hNIL AAVS1 3’ Junction F | GCCTGGTAGACAGGGCTGG |
| hNIL AAVS1 3’ Junction R | TGTGGGGTGGAGATATCAGC |
| AAVS1 WT F | CGGTTAATGTGGCTCTGGTT |
| AAVS1 WT R | AGGATCCTCTCTGGCTCCAT |
| hNIL CLYBL 5’ Junction F | CAGACAAGTCAGTAGGGCCA |
| hNIL CLYBL 5’ Junction R | AGAAGACTTCCTCTGCCCTC |
| hNIL CLYBL 3’ Junction F | CACCAGCAACCTGACGTTTT |
| hNIL CLYBL 3’ Junction R | TTTTATAGGCGCCCACCGTA |
| CLYBL WT F | TGACTAAACACTGTGCCCCA |
| CLYBL WT R | AGGCAGGATGAATTGGTGGA |
| N98S Sequencing F | TACTCGACCTCCTACAAGCG |
| N98S Sequencing R | GCGGCTCTTGAACCATTCCT |
| ssODN for HDR | CACGCGCTCGATGAAGCTGGCGAAGCGATCATTGAGGTCCTGGAGCTGCGCCTTCTCCTG |
| Off-target 1 F | AAAAGTGTTGGTGCAGATGTGG |
| Off-target 1 R | TGGTGGACATTACGCACAGA |
| Off-target 2 F | AAAAGTGTTGGTGCAGATGTGG |
| Off-target 2 R | TGTCAATGGATGCTTGGGTGG |
| Off-target 3 F | TCACCAATTCATGGGACGCC |
| Off-target 3 R | AAACTCCCACTCACGGCAAG |
| Off-target 4 F | GGCAGTCAAAACCCCATCCT |
| Off-target 4 R | CCTGGTGGGACAAGGTGAAA |
| Off-target 5 F | CCCTGGTATTGGCAGTTTAGC |
| Off-target 5 R | TGCCCTCCTAACTGAACATTCC |
| Off-target 6 F | TATGTGTCCCGTTTCCTCGG |
| Off-target 6 R | AGACAGACTACGGCAGGAGC |
| Off-target 7 F | GAACCATGGAGACCTCACCG |
| Off-target 7 R | GGCCAGCAGACAGACTATGG |
| Off-target 8 F | AAGAGGAGAACCACATGGGC |
| Off-target 8 R | GACAGGAGACCTGGAGTTACG |
| Off-target 9 F | GTTGGGTCAGCTCTCTTCTCG |
| Off-target 9 R | GATGGAAAGCCTGGAGTTGC |
| Off-target 10 F | CACTTTCCCTTGGATCTGCCT |
| Off-target 10 R | TCATTGGCAGCAGAGAACACT |
| Off-target 11 F | AGCCTCAGCAAATGATGGCA |
| Off-target 11 R | ATGATTCCCGCAGGTCACTG |

**Supplementary Table 2: Guide RNA Sequences**

| **Name** | **gRNA spacer sequence** |
| --- | --- |
| N98S Sp.HiFi | AGCTGGCGAAGCGGTCACTG |
| N98S Sa.KKH | TGAAGCTGGCGAAGCGGTCAT |

**Supplementary Table 3: Antibodies**

| **Name/Antigen** | **Host** | **Vendor/Catalog #** | **IF Concentration** | **WB Concentration** |
| --- | --- | --- | --- | --- |
| β-Tubulin III | Rabbit | Sigma T2200 | 1:500 |  |
| SMI33 (NF-H/M) | Mouse | Biolegend 835404 | 1:500 |  |
| HB9 | Mouse | DSHB 81.5C10 | 1:200 |  |
| NF-L | Rabbit | Sigma AB9568 | 1:500 |  |
| NF-L | Mouse | Sigma N5139 |  | 1:1000 |
| GAPDH | Rabbit | Abcam AB9485 |  | 1:2000 |
| Anti-rabbit  Alexa Fluor 488 | Goat | Invitrogen A11008 | 1:500 |  |
| Anti-mouse  Alexa Fluor 594 | Goat | Invitrogen A11005 | 1:500 |  |
| Anti-rabbit  IRDye 800CW | Donkey | Li-Cor 926-32213 |  | 1:10,000 |
| Anti-mouse IRDye 680LT | Donkey | Li-Cor 926-68022 |  | 1:10,000 |

**Supplementary Table 4: Gene Expression Assay IDs**

| **Gene Target** | **Vendor** | **Primer/Probe Assay ID** |
| --- | --- | --- |
| *MNX1* | ThermoFisher | Hs00907365_m1 |
| *CHAT* | ThermoFisher | Hs00758143_m1 |
| *NEFL* | ThermoFisher | Hs01034882_m1 |
| *GAPDH* | Bio-Rad | qHsaCEP0041396 |

**Supplementary Table 5: Genomic off-target analysis**

| **#** | **chr** | **start (hg38)** | **End (hg38)** | **Strand** | **CHD score** | **Description** | **Results** |
| --- | --- | --- | --- | --- | --- | --- | --- |
| 1 | 1 | 148316436 | 148316458 | + | 0.7143 | intergenic:  RP11-495P10.8/RP11-495P10.5/RP11-495P10.7-RP11-495P10.3 | No mutations |
| 2 | 12 | 5752303 | 5752303 | - | 0.4412 | intron: ANO2 | Sequencing failed |
| 3 | 10 | 75661072 | 75661094 | - | 0.3956 | intergenic:  RP11-310J24.3-RP11-367B6.2 | No mutations |
| 4 | 20 | 48137627 | 48137649 | + | 0.3652 | intergenic:  AL139351.1-LINC00494 | No mutations |
| 5 | 18 | 39020019 | 39020041 | - | 0.3649 | intergenic:  RN7SKP182-RNU6-706P | No mutations |
| 6 | 4 | 3605528 | 3605550 | - | 0.3563 | intergenic:  LINC00955-RP3-368B9.2 | No mutations |
| 7 | 9 | 65426450 | 65426472 | + | 0.3563 | intergenic:  FOXD4L5-Y_RNA | No mutations |
| 8 | 8 | 1925519 | 1925541 | - | 0.3545 | intron: ARHGEF10 | No mutations |
| 9 | 13 | 111494882 | 111494904 | + | 0.3254 | intergenic:  TEX29-RP11-65D24.2 | Het. -/G rs34866221 |
| 10 | 8 | 120892179 | 120892201 | - | 0.3196 | intergenic:  RP11-713M15.2/SNTB1-RP11-369K17.1 | No mutations |
| 11 | 9 | 14223924 | 14223946 | + | 0.2790 | intron: NFIB | No mutations |
